# Rotavirus vaccine coverage and factors associated with uptake using linked data: Ontario, Canada

**DOI:** 10.1371/journal.pone.0192809

**Published:** 2018-02-14

**Authors:** Sarah E. Wilson, Hannah Chung, Kevin L. Schwartz, Astrid Guttmann, Shelley L. Deeks, Jeffrey C. Kwong, Natasha S. Crowcroft, Laura Wing, Karen Tu

**Affiliations:** 1 Public Health Ontario, Toronto, Ontario, Canada; 2 Institute for Clinical Evaluative Sciences, Toronto, Ontario, Canada; 3 Dalla Lana School of Public Health, University of Toronto, Toronto, Ontario, Canada; 4 Department of Pediatrics, Hospital for Sick Children, Toronto, Ontario, Canada; 5 Institute of Health Policy, Management and Evaluation, University of Toronto, Toronto, Ontario, Canada; 6 Department of Family and Community Medicine, University of Toronto, Toronto, Ontario, Canada; 7 Department of Laboratory Medicine and Pathobiology, University of Toronto, Toronto, Ontario, Canada; Johns Hopkins Bloomberg School of Public Health, UNITED STATES

## Abstract

**Background:**

In August 2011, Ontario, Canada introduced a rotavirus immunization program using Rotarix^™^ vaccine. No assessments of rotavirus vaccine coverage have been previously conducted in Ontario.

**Methods:**

We assessed vaccine coverage (series initiation and completion) and factors associated with uptake using the Electronic Medical Record Administrative data Linked Database (EMRALD), a collection of family physician electronic medical records (EMR) linked to health administrative data. Series initiation (1 dose) and series completion (2 doses) before and after the program’s introduction were calculated. To identify factors associated with series initiation and completion, adjusted odds ratios (aOR) and 95% confidence intervals (95%CI) were calculated using logistic regression.

**Results:**

A total of 12,525 children were included. Series completion increased each year of the program (73%, 79% and 84%, respectively). Factors associated with series initiation included high continuity of care (aOR = 2.15; 95%CI, 1.61–2.87), maternal influenza vaccination (aOR = 1.55; 95%CI,1.24–1.93), maternal immmigration to Canada in the last five years (aOR = 1.47; 95% CI, 1.05–2.04), and having no siblings (aOR = 1.62; 95%CI,1.30–2.03). Relative to the first program year, infants were more likely to initiate the series in the second year (aOR = 1.71; 95% CI 1.39–2.10) and third year (aOR = 2.02; 95% CI 1.56–2.61) of the program. Infants receiving care from physicians with large practices were less likely to initiate the series (aOR 0.91; 95%CI, 0.88–0.94, per 100 patients rostered) and less likely to complete the series (aOR 0.94; 95%CI, 0.91–0.97, per 100 patients rostered). Additional associations were identified for series completion.

**Conclusions:**

Family physician delivery achieved moderately high coverage in the program’s first three years. This assessment demonstrates the usefulness of EMR data for evaluating vaccine coverage. Important insights into factors associated with initiation or completion (i.e. high continuity of care, smaller roster sizes, rural practice location) suggest areas for research and potential program supports.

## Introduction

Prior to the implementation of vaccination programs, rotavirus was a common cause of childhood gastroenteritis, responsible for up to 40% of acute gastroenteritis presentations (depending on season) and a cause of substantial healthcare utilization [1,2]. In the pre-vaccine era in Canada, one-third of children with rotavirus gastroenteritis sought care in an outpatient setting, 15% used emergency department services and 7% required hospitalization [2]. Two live attenuated oral rotavirus vaccines are authorized for use in Canada: RotaTeq^®^ (RV5, Merck Canada Inc.) since 2006 [3] and Rotarix^™^ (RV1, GlaxoSmithKline Inc.) as of 2007[4]. Canada’s National Advisory Committee on Immunization (NACI) issued recommendations for the use of rotavirus vaccines in 2008 and 2010[5,6]. In August 2011, Ontario implemented a universal publicly-funded rotavirus immunization program with RV1 vaccine at 2 and 4 months of age. Prior to the program, parents could purchase the vaccine with a physician prescription. The publicly-funded program has been associated with a 71% reduction in hospitalizations due to rotavirus infection [7]. However, a formal coverage evaluation has been challenged by two issues. First, the routine processes for coverage monitoring in Ontario delay assessment until the time of school entry. Second, physicians are not remunerated for the delivery of this oral vaccine (in contrast to parenteral vaccines); consequently there is no immunization delivery billing code available in health administrative data. The use of electronic medical records (EMRs) may help fill this information gap.

The objectives of this study were to: (1) assess rotavirus vaccine coverage in Ontario using EMR data as recorded in family physician offices; (2) assess compliance with age-based vaccine administration recommendations; and (3) identify factors associated with series initiation and completion.

## Methods

### Study population and setting

In Ontario, Canada’s most populous province (population 13.5 million in 2013)[8], infant and toddler immunizations are almost exclusively administered through physician offices, by family physicians, pediatricians and nurse practitioners. The majority of pediatric primary care health services are delivered by family physicians [9]. Medical services, including immunization delivery, are funded by the universal, single-payer Ontario Health Insurance Plan (OHIP).

We included pre-defined birth cohorts who receive primary care from family physicians who share their data with the Electronic Medical Record Administrative data Linked Database (EMRALD), a centralized repository of EMR data. EMRALD has been formally evaluated [10,11] and used for research using EMR data alone or, after linkage to health administrative data [12–16]. It has also been used to validate the introduction of vaccine-specific billing codes in Ontario [17]. That study found that the data within EMRALD was more complete (i.e. had a greater number of immunization events) compared to billing claims data [17]. EMRALD contains EMR data from over 350 Ontario family physicians using PS Suite^®^ EMR software, the most widely used EMR platform in Ontario. This represents approximately 3% of practicing family physicians [18]. Individual-level data from EMRALD are collected annually and linked to health administrative databases at the Institute for Clinical Evaluative Sciences (ICES).

We created six cohorts of children to evaluate coverage during the first three years of the program and to assess coverage during the preceding years when the vaccine was recommended by NACI, but not publicly-funded (i.e. not included within Ontario’s routine immunization schedule). Children with birth dates between August 1, 2011 and July 31, 2014 and who received care from an EMRALD physician who had submitted EMR data during the most recent round of data collection (summer of 2015) were included in the coverage assessment of the program period. Children born between January 1, 2008 and December 31, 2010 were included in the coverage assessment of privately purchased vaccine. We used the date of birth range of January 1 to July 31, 2011 as a wash-out period as children born during this period may have started the series with privately purchased vaccine and completed it as part of the public program.

We excluded children with fewer than 3 visits recorded in EMRALD, those who died during the first year of life, and those with an EMR start date >6 weeks after birth. We also excluded children with multiple identification numbers, missing sex or birthdate information, or in cases where there was discrepant demographic information between EMRALD and Ontario’s Registered Persons Database.

### Data sources

#### Rotavirus immunization status

We conducted text searches for rotavirus vaccine using generic terms and proprietary names in the immunizations field of the continuous patient profile (CPP) and in the “treatments/prescriptions” fields using a series of keywords (e.g., “rotavirus”, “Rotarix”, “Rotateq”). Most entries use structured terms but because free-text data entry is also possible, search terms also included keywords with spelling errors (e.g. “Rotarx”). S1 Appendix outlines the complete list of search terms. If multiple doses were recorded as administered on the same date, we assumed this reflected data entry error and only one dose was used for analyses. We assessed the completeness of our EMR search methodology to identify rotavirus immunization events in a post -hoc analysis, outlined further in S2 Appendix.

#### Covariates

Several health administrative databases housed at ICES were linked using unique encoded identifiers to obtain covariate information for the cohort child, their mother and assigned physician.

#### Infant characteristics

We used Ontario’s Registered Persons Database (RPDB) to identify the child’s sex, vital statistics, and postal code. The RPDB is a population-based repository of demographic information including unique health card number for all Ontario residents who are eligible for health services under OHIP. Postal code was linked to 2006 Statistic Canada Census postal code information to determine: (1) rural residence (community size < 10,000) and (2) mean household income quintile (adjusted for household and community size) of the enumeration or dissemination area as a proxy for socioeconomic status.

The MOMBABY database is comprised of admission records of delivering mothers and their newborn babies which are linked through a unique matching number on each hospitalization record. This dataset was used to identify low birth weight (< 2500 grams) and premature (< 37 weeks gestation) infants. We used the Canadian Institute of Health Information’s Discharge Abstract Database (CIHI-DAD) to identify chronic medical conditions among children, as described by Feudtner et al. [19] and those with congenital malformations and/or chromosomal abnormalities (using International Classification of Diseases-10 (ICD-10) codes Q00-Q99) in hospitalizations during the first year of life.

We defined receipt of other childhood vaccines as having received at least one dose of the multicomponent diphtheria, tetanus, acellular pertussis, inactivated polio, and *Haemophilus influenzae* type b (DTaP-IPV-Hib) vaccine or pneumococcal conjugate vaccine (PCV) administered between 6 weeks and 4 months of age, assessed in days (42–112 days). We accepted either vaccine documentation in EMRALD or the presence of a vaccine-specific OHIP billing code for administration (DTaP-IPV-Hib = G841 and PCV = G846).

OHIP data was also used to identify the number of primary care visits in the first year of life and to calculate a continuity of care (COC) score. COC is defined as the number of visits to an individual’s usual primary care physician divided by the total number of primary care visits during the first year of life. We defined a COC of less than 50% as low [20,21]. For the study cohort, the usual primary care physician was defined by physician information within EMRALD. For the Ontario reference cohort presented in Table 1, children were assigned to a usual primary care physician based on roster data within the Client Agency Program Enrolment (CAPE). If a child was not rostered, he or she was assigned to the physician who billed the most primary care OHIP visits in the first year of life, based on total cost (primary care codes available upon request).

**Table 1:** Child and maternal characteristics of the EMRALD study cohorts as compared to the 2013 Ontario birth cohort.

VARIABLE	VALUE	Ontario 2013 Birth Cohort	EMRALD Private period	EMRALD Program period	Standardized Difference
		N = 131,206	N = 5,039	N = 7,486	
**Child’s characteristics**					
Sex	Male	67,214 (51.2%)	2,605 (51.7%)	3,786 (50.6%)	0.00
	Female	63,992 (48.8%)	2,434 (48.3%)	3,700 (49.4%)	0.00
Rural residence	Yes	12,927 (9.9%)	1,075 (21.3%)	1,095 (14.6%)	0.22
	No	113,043 (86.2%)	3,949 (78.4%)	6,162 (82.3%)	0.15
	Missing information	5,236 (4.0%)	15 (0.3%)	229 (3.1%)	0.12
Income quintile	1 (Lowest)	28,131 (21.4%)	885 (17.6%)	1,423 (19.0%)	0.08
	2	25,392 (19.4%)	1,050 (20.8%)	1,336 (17.8%)	0.01
	3	25,212 (19.2%)	1,125 (22.3%)	1,532 (20.5%)	0.05
	4	26,251 (20.0%)	1,065 (21.1%)	1,607 (21.5%)	0.03
	5 (Highest)	20,372 (15.5%)	882 (17.5%)	1,329 (17.8%)	0.06
	Missing information	5,848 (4.5%)	32 (0.6%)	259 (3.5%)	0.12
Any chronic condition diagnosed in the 1st year of life or congenital malformation		6,860 (5.2%)	162 (3.2%)	316 (4.2%)	0.07
Low birth weight (<2500 g)	Yes	8,272 (6.3%)	217 (4.3%)	366 (4.9%)	0.07
	No	122,931 (93.7%)	4,718 (93.6%)	6,998 (93.5%)	0.01
	Missing information	< = 5 (0.0%)	104 (2.1%)	122 (1.6%)	0.19
Preterm at delivery (<37 weeks)	Yes	10,220 (7.8%)	344 (6.8%)	464 (6.2%)	0.05
	No	120,935 (92.2%)	4,589 (91.1%)	6,896 (92.1%)	0.02
	Missing information	51 (0.0%)	106 (2.1%)	126 (1.7%)	0.19
Number of primary care visits (OHIP) in the 1st year of life	Mean (SD)	9.28 (4.88)	9.21 (3.76)	9.11 (3.61)	0.03
	Median (IQR)	9 (6–12)	9 (7–11)	9 (7–11)	0.01
Continuity of care	Low (<50%)	18,436 (14.1%)	908 (18.0%)	1,509 (20.2%)	0.14
	High (= >50%)	109,350 (83.3%)	4,115 (81.7%)	5,968 (79.7%)	0.07
	No primary care visits in 1^st^ year of life	3,420 (2.6%)	16 (0.3%)	9 (0.1%)	0.21
**Mother’s characteristics**					
Recent immigrant (landing date within 5 years of child’s birth)	Yes	12,112 (9.2%)	289 (5.7%)	565 (7.5%)	0.09
	No	115,779 (88.2%)	4,580 (90.9%)	6,665 (89.0%)	0.05
	Missing information	3,315 (2.5%)	170 (3.4%)	256 (3.4%)	0.05
Maternal age at first pregnancy	<24	34,788 (26.5%)	1,370 (27.2%)	1,758 (23.5%)	0.04
	25–34	78,463 (59.8%)	2,912 (57.8%)	4,477 (59.8%)	0.02
	35+	14,640 (11.2%)	587 (11.6%)	995 (13.3%)	0.05
	Missing information	3,315 (2.5%)	170 (3.4%)	256 (3.4%)	0.05
Number of children at time of birth of cohort child (including cohort child)	1	61,876 (47.2%)	2,375 (47.1%)	3,617 (48.3%)	0.01
	2	43,828 (33.4%)	1,685 (33.4%)	2,489 (33.2%)	0.00
	3+	22,132 (16.9%)	809 (16.1%)	1,124 (15.0%)	0.04
	Missing information	3,370 (2.6%)	170 (3.4%)	256 (3.4%)	0.05
Influenza vaccination in year following delivery	Yes	22,282 (17.0%)	885 (17.6%)	1,422 (19.0%)	0.04
	No	105,609 (80.5%)	3,984 (79.1%)	5,808 (77.6%)	0.06
	Missing information	3,315 (2.5%)	170 (3.4%)	256 (3.4%)	0.05

Standardized differences were calculated to compare the characteristics of the EMRALD cohort (private and program periods combined) with the Ontario 2013 birth cohort.

#### Maternal and family characteristics

We used the MOMBABY database and the CIHI-DAD to identify the mothers of the children in the study cohort and to determine maternal age at first delivery. MOMBABY was also used to determine the number of children in the household, based on the number of prior deliveries of the study cohort’s biological mother (i.e. previous stillbirths were excluded from the count).

We used the Immigration, Refugees and Citizenship Canada (IRCC) Permanent Resident database, containing information on individuals who have landed in Ontario since 1985, to determine recent maternal immigration; recent was defined by a landing date within the 5 years prior to the cohort child’s birth.

Finally, we used OHIP and Ontario Drug Benefit (ODB) databases to identify maternal influenza immunization during the year following delivery. The influenza-specific OHIP billing codes of G590 and G591 and the drug identification numbers (DIN#s) ‘02346850’, ‘02223929’, ‘02362384’, ‘02015986’ from ODB were used.

#### Physician characteristics

The ICES Physician Database contains information on physician demographics and specialization. We obtained the following physician information: sex, rural practice, decade of graduation, and place of undergraduate medical training (Canada vs. outside of Canada). We used the CAPE database to determine total patient roster size effective July 1, 2013, by physician.

### Analysis

#### Descriptive epidemiology of the study cohort and their physicians

We compared the characteristics of our study cohort (private and public program periods combined) with the 2013 Ontario birth cohort and completed a similar comparison of EMRALD physicians represented in our study with the primary care providers (family physicians and pediatricians) for the reference birth cohort. We calculated standardized differences, a measure that is not as sensitive to sample size as traditional tests [22]. Standardized differences of greater than 0.1 are considered to be meaningful [23].

#### Coverage

We defined full series coverage as 2 doses of RV1 or 3 doses of RV5. If a series of mixed products was used, 3 doses were required for completion [24]. If rotavirus vaccine was recorded without a trade name, it was assumed to be RV1. Coverage was calculated using two approaches. The first involved counting all immunization events, regardless of the timing between doses, whereas the second approach considered only doses that were separated by the recommended minimum interval (4 weeks). Cochrane-Armitage trend tests were used to determine whether there was a linear trend in coverage over time. We conducted a sensitivity analyses to explore if altering our inclusion criterion of requiring at least 3 EMR visits in the first year of life impacts immunization coverage.

#### Compliance

We assessed compliance for birth cohorts eligible for publicly-funded RV1 vaccine. Children who received Rotateq^®^ were excluded as our primary interest was implementation of the publicly-funded program. We assessed adherence to the age-based recommendations outlined in the Canadian Immunization Guide (CIG) [24] and RV1 product monograph [4] separately. As with coverage, we made the assumption that children for whom generic rotavirus vaccine was recorded in EMRALD received RV1.

#### Multivariable regression

We assessed associations between covariates and series initiation and completion among the birth cohorts eligible for publicly-funded vaccine using logistic regression with the use of general estimating equations to account for clustering at the physician level. We excluded children with missing covariate information and patients assigned to one physician who did not initiate the series, as inclusion of these children (n = 39) caused errors in running the model. We assessed series initiation and completion separately. We accepted any first dose for our measure of series initiation and did not apply a minimum interval in defining series completion as we were interested in factors associated with vaccine uptake, rather than factors associated with children receiving a perfect schedule. Covariates were identified *a priori* for inclusion in each model and informed by Canadian rotavirus coverage research priorities [25], other studies in the coverage literature [26–30] and supplemented with additional factors we felt operationalized domains relevant to the World Health Organization vaccine acceptance framework of: Complacency, Convenience and Confidence [31]. These included characteristics of the infant (sex, rural residence, neighbourhood income quintile, comorbidity, low birth weight, prematurity, number of primary care visits during the first year of life, continuity of care (> = 50% of visits to the same primary care physician), receipt of the first dose of DTaP-IPV-Hib vaccine or PCV; mother (recent immigration, age at first pregnancy, influenza vaccination in the year following delivery of the cohort child, number of siblings at the time of the cohort child’s birth); and physician (sex, rural practice, decade of graduation from medical school, foreign training, total patient roster size). Program year was included in the model to account for possible trends in program delivery over time (e.g., increased familiarity of providers with the vaccine program). Receipt of other childhood vaccinations and maternal influenza vaccination were hypothesized to be more relevant to initiation and were not included in the model examining series completion. We present unadjusted and adjusted odds ratios (aOR) and 95% confidence intervals (95% CI) for each covariate. All p-values are two-sided.

#### Ethics, consent and privacy statements

This study was approved by the institutional review boards at Sunnybrook Health Sciences Centre and Public Health Ontario in Toronto, Canada. Similar to other studies using administrative data, subjects were not contacted to receive individual expressed consent. However, all analyses occurred following de-identification. Datasets were linked using unique encoded identifiers and analyzed at ICES using SAS software, version 9.4 (SAS Institute, Cary, NC).

## Results

**Fig 1:**
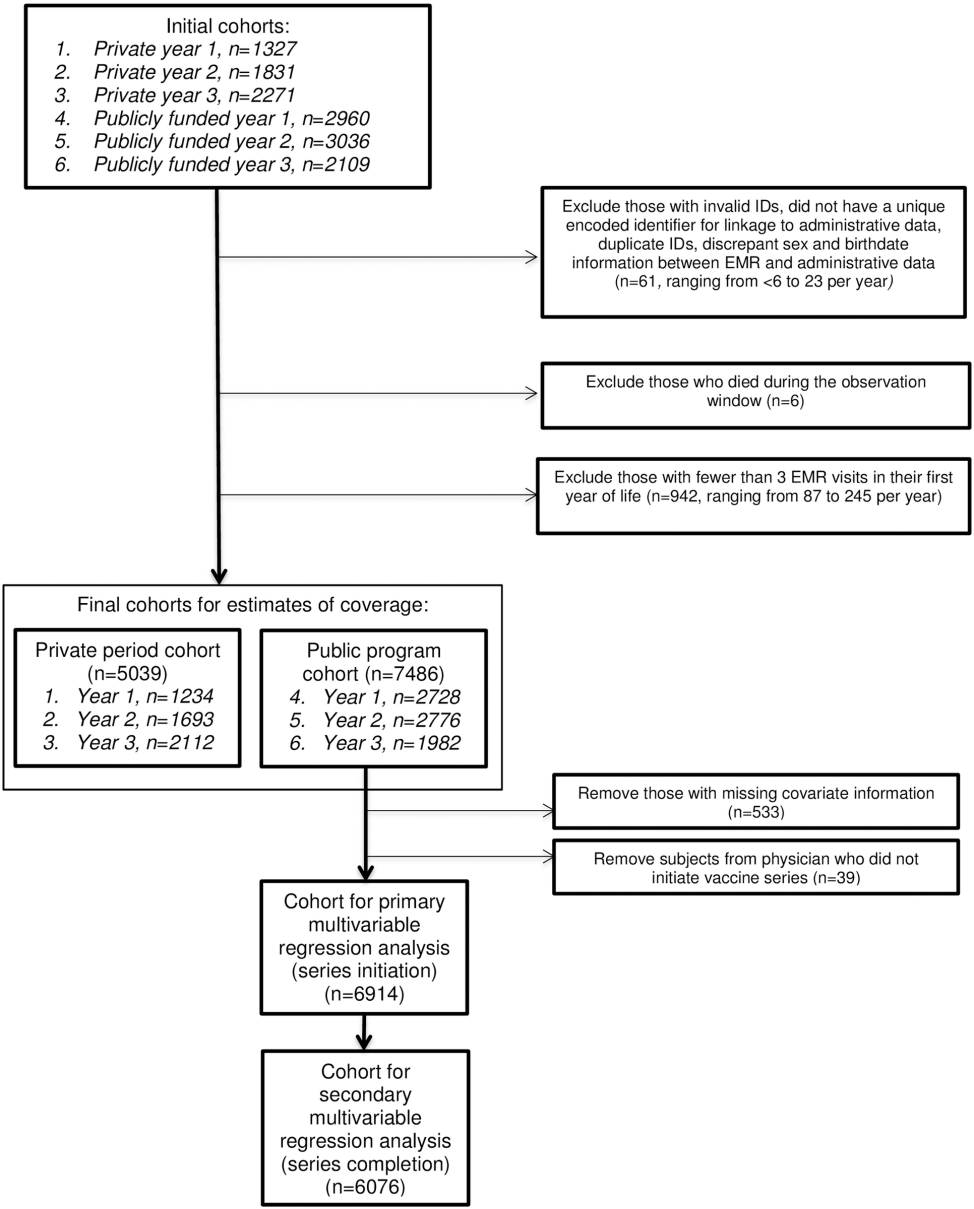
Flow diagram of exclusions made to produce the final cohort for descriptive and multivariable analyses (please see attachment).

We identified 13,534 children who were born between January 1, 2008 to December 31, 2010, and August 1, 2011 to July 31, 2014 in EMRALD, of which 99% were successfully linked to administrative databases. After study exclusions, there were 12,525 children included in our coverage assessment: 5,039 born during the period of private vaccine eligibility and 7,486 eligible for publicly-funded vaccine (Fig 1). Characteristics of the study children and their mothers were compared to the 2013 Ontario birth cohort (Table 1). The two groups were similar across most characteristics with very few exceptions, as assessed by standardized differences. A greater proportion of study children lived in rural areas and had lower continuity of care. The 335 unique EMRALD family physicians providing care to study children were more likely to be women, less likely to be foreign-trained, with smaller roster sizes and with fewer years in practice compared to the usual primary care providers (family physicians and pediatricians) seen by the 2013 Ontario birth cohort (Table 2), as assessed using standardized differences.

**Table 2:** EMRALD study cohort physician characteristics compared to reference physicians who provide primary care to the 2013 Ontario birth cohort.

VARIABLE	VALUE	Ontario Primary Care Physicians for 2013 Cohort	EMRALD Physicians for Study Cohort	Standardized Difference
		N = 8,748	N = 335	
Physician’s (UPC) Characteristics				
Sex	Male	4,749 (54.3%)	145 (43.3%)	0.22
	Female	3,947 (45.1%)	184 (54.9%)	0.20
	Missing information	52 (0.6%)	6 (1.8%)	0.11
Rural practice	Yes	820 (9.4%)	40 (11.9%)	0.08
	No	7,856 (89.8%)	288 (86.0%)	0.12
	Missing information	72 (0.8%)	7 (2.1%)	0.11
Foreign trained	Yes	2,756 (31.5%)	31 (9.3%)	0.57
	No	5,930 (67.8%)	298 (89.0%)	0.53
	Missing information	62 (0.7%)	6 (1.8%)	0.10
Decade of graduation from medical school	1960-70s	1,630 (18.6%)	39 (11.6%)	0.20
	1980s	2,475 (28.3%)	56 (16.7%)	0.28
	1990s	2,178 (24.9%)	84 (25.1%)	0.00
	2000s	2,413 (27.6%)	150 (44.8%)	0.36
	Missing information	52 (0.6%)	6 (1.8%)	0.11
Number of patients rostered as of July 1, 2013	Mean (SD)	1,051 (870)	937 (609)	0.15
	Median (IQR)	1,022 (56–1,608)	879 (502–1,355)	0.11

Series coverage for infants born during the period of private purchase ranged from 3.0% to 7.2% (Table 3). Among the birth cohorts eligible for publicly-funded vaccine, series initiation ranged from 83.2% to 91.3%. Full series coverage significantly increased each year of the program, from 73.0%, to 78.5% and 84.2% (test for trend, p< 0.0001). Series completion among initiators also increased over time, and was higher in the public program period. Full series coverage decreased by 0.2% or less if coverage was calculated requiring a minimum interval of 28 days between doses. Our sensitivity analysis with modified inclusion criterion allowing fewer EMR visits resulted in minimal changes to the immunization coverage results. During the private purchase period, series coverage estimates were reduced by 0.4% or less, if the inclusion criteria were changed to require only one EMR visit. Series coverage estimates during the public program were reduced by 2.3% or less if a similar modification was made and by 4.0% or less if there was no EMR visit criterion factored into the creation of the analytic cohort (S3 Appendix).

**Table 3:** Rotavirus vaccine initiation and full series coverage, by birth cohort and program status.

Program status	Birth cohort	Cohort size	Proportion of children who initiated the rotavirus vaccine series^1^ (with 95% CI)	Proportion of initiators who completed series^2^ (all doses considered) (with 95% CI)	Coverage estimate for series completion^2^ (all doses considered) (with 95% CI)
Private availability	Jan. 1-Dec. 31, 2008	1234	4.9% (3.7%, 6.1%)	61.7% (49.4%, 74.0%)	3.0% (2.0%, 3.8%)
Private availability	Jan. 1-Dec. 31, 2009	1693	8.1% (6.8%, 9.4%)	64.2% (56.2%, 72.2%)	5.2% (4.1%, 6.3%)
Private availability	Jan. 1-Dec. 31, 2010	2112	11.0% (9.7%, 12.3%)	65.2% (59.1%, 71.3%)	7.2% (5.9%, 8.1%)
Program year 1	Aug. 1 2011-July 31, 2012	2728	83.2% (81.8%, 84.6%)	87.7% (86.3%, 89.1%)	73.0% (71.1%, 74.5%)
Program year 2	Aug. 1 2012-July 31, 2013	2776	89.0% (87.8%, 90.2%)	88.2% (86.9%, 89.5%)	78.5% (76.9%, 79.9%)
Program year 3	Aug. 1 2013-July 31, 2014	1982	91.3% (90.1%, 92.5%)	92.2% (91.0%, 93.4%)	84.2% (82.4%, 85.6%)

^1^Receipt of at least one dose of rotavirus vaccine (RV1 or RV5).

^2^Series completion required two doses if all the doses were RV1. Three doses were required if all doses were RV5 or if a mix of products (RV1 and RV5) were used.

Compliance with age-based scheduling recommendations for publicly-funded rotavirus vaccine was high (Table 4). Nearly all subjects received the vaccine on/after 6 weeks of age (>99%) with a minimum interval of 4 weeks between doses (>99%). Among the 5,832 children who received at least two doses of RV1, 97.3% received the final dose of a multi-dose series on or before 24 weeks of age, as per product monograph. It was administered on or before 32 weeks of age (as per CIG guidance) in 99.7%. Three doses of RV1 or a generic term for rotavirus vaccine were documented in the EMR among twenty-six children in the public program period (<1%).

**Table 4:** Compliance and non-compliance with vaccine administration recommendations during the program period (n = 6527)^a^^,^^b^.

	Guidance document	Yes	Denominator	%
A	**Recommendations outlined in the Canadian Immunization Guide (CIG)**			
	First dose < 15 weeks of age	6141	6527	94.1
B	Final dose of a multi-dose series administered < 32 weeks of age	5817	5832^b^	99.7
	**Recommendations outlined in the RV1 product monograph**			
C	First dose < 20 weeks of age	6476	6527	99.2
D	Final dose of a multi-dose series administered < 24 weeks of age	5676	5832^b^	97.3
	**Consistent recommendations in both the CIG and RV1 product monograph**			
E	First dose at > = 6 weeks of age	6521–6527^c^	6527	>99.0
F	Minimum interval of 4 weeks between doses	5819	5832^b^	99.8
G	Maximum of two doses for series completion	5806	5832	99.6
	**Total measure of compliance with recommendations**			
	Using CIG age-based parameters (compliance = A+B+E+F+G)	5731	6527	87.8
	Using RV1 product monograph age-based parameters (compliance = C+D+E+F+G)	5658	6527	86.7

^a^Children who received at least one dose of Rotateq during the program period were excluded from this analysis.

^b^5832 children received at least 2 doses of rotavirus vaccine (i.e. RV1 or generic identification in EMRALD during the program period). A total of 26 children received 3 doses.

^c^A range is presented to suppress disclosure of a small cell size (< 6 subjects)

Factors significantly associated with the odds of series initiation in multivariable modelling included high continuity of care (aOR = 2.15; 95%CI,1.61–2.87), maternal influenza vaccination in the year following delivery (aOR = 1.55; 95%CI,1.24–1.93), maternal immigration to Canada in the previous five years (aOR = 1.47, 95% CI 1.05–2.04), and having no siblings (aOR = 1.62; 95%CI,1.30–2.03) or only one sibling (aOR = 1.31; 95%CI, 1.04–1.65)(Table 5). Relative to the first program year, infants were more likely to initiate the series in the second year (aOR = 1.71; 95% CI 1.39–2.10) and third year (aOR = 2.02; 95% CI 1.56–2.61) of the program. Infants were less likely to initiate the series if they had not received other routine infant vaccines (aOR = 0.04; 95%CI, 0.03–0.07) or received care from a physician with a larger patient roster size (aOR 0.91; 95%CI, 0.88–0.94, per 100 patients rostered). Among initiators, those with a greater number of primary care visits in the first year of life: 7–11 visits (aOR = 1.61; 95%CI, 1.30–1.99), > = 12 visits (aOR = 1.43; 95%CI,1.10–1.85), high continuity of care (aOR = 1.61; 95%CI, 1.27–2.04), or receiving care from a physician in rural practice (aOR = 2.08; 95%CI, 1.29–3.35) were more likely to complete the series. Infants were also more likely to complete the series if they initiated the vaccine in the third program year (aOR = 1.49; 95% CI 1.15–1.92), relative to the first year of the program. Children born to a mother who was younger than 24 years of age at the time of her first pregnancy (aOR = 0.71; 95%CI, 0.58–0.86), and those receiving care from a physician trained outside of Canada (aOR = 0.60; 95%CI, 0.39–0.92) and from physicians with a larger patient roster size (aOR 0.94; 95%CI, 0.91–0.97, per 100 patients rostered) were less likely to complete the series. There was no association between series initiation or completion and neighbourhood income quintile, rural residence, prematurity or low birthweight.

**Table 5:** Unadjusted and adjusted ORs of rotavirus (RV) vaccine series initiation and series completion among children born during the public program years.

Characteristic		Series initiation (n = 6914)		Series completion among initiators^1^ (n = 6076)	
		Unadjusted OR	Adjusted OR	Unadjusted OR	Adjusted OR
		(95% CI)	(95% CI)	(95% CI)	(95% CI)
**Program characteristics**					
Program year	Year 1	1.00 (ref)	1.00 (ref)	1.00 (ref)	1.00 (ref)
	Year 2	1.59 (1.29–1.97)	1.71 (1.39–2.10)	1.18 (0.96–1.44)	1.13 (0.93–1.39)
	Year 3	2.02 (1.54–2.65)	2.02 (1.56–2.61)	1.56 (1.20–2.03)	1.49 (1.15–1.92)
**Child’s characteristics**					
Sex	Female	1.00 (ref)	1.00 (ref)	1.00 (ref)	1.00 (ref)
	Male	1.03 (0.90–1.19)	1.07 (0.94–1.22)	1.05 (0.89–1.25)	1.06 (0.90–1.24)
Rural residence	No	1.00 (ref)	1.00 (ref)	1.00 (ref)	1.00 (ref)
	Yes	0.78 (0.61–0.99)	0.87 (0.69–1.09)	1.32 (0.99–1.76)	1.20 (0.89–1.61)
Income quintile	1 (Lowest)	1.00 (ref)	1.00 (ref)	1.00 (ref)	1.00 (ref)
	2	0.86 (0.69–1.07)	0.86 (0.68–1.08)	1.14 (0.88–1.48)	1.07 (0.82–1.38)
	3	0.90 (0.72–1.12)	0.95 (0.78–1.17)	1.13 (0.85–1.50)	1.05 (0.79–1.39)
	4	1.00 (0.81–1.23)	1.03 (0.84–1.26)	1.19 (0.94–1.52)	1.08 (0.85–1.37)
	5 (Highest)	0.84 (0.66–1.06)	0.85 (0.67–1.07)	1.49 (1.14–1.95)	1.31 (0.99–1.73)
Comorbidities	No	1.00 (ref)	1.00 (ref)	1.00 (ref)	1.00 (ref)
	Yes	1.06 (0.74–1.50)	1.27 (0.87–1.87)	0.82 (0.56–1.21)	0.82 (0.57–1.19)
Low birth weight	No	1.00 (ref)	1.00 (ref)	1.00 (ref)	1.00 (ref)
	Yes	1.26 (0.87–1.82)	1.32 (0.91–1.91)	1.04 (0.71–1.51)	1.09 (0.73–1.62)
Prematurity	No	1.00 (ref)	1.00 (ref)	1.00 (ref)	1.00 (ref)
	Yes	0.96 (0.69–1.34)	0.90 (0.62–1.31)	1.03 (0.72–1.46)	0.98 (0.67–1.43)
Number of PC OHIP visits in 1st year of life	0–6	1.00 (ref)	1.00 (ref)	1.00 (ref)	1.00 (ref)
	7–11	1.37 (1.14–1.65)	0.98 (0.84–1.14)	1.67 (1.34–2.06)	1.61 (1.30–1.99)
	12+	1.26 (1.01–1.58)	0.88 (0.70–1.09)	1.42 (1.11–1.83)	1.43 (1.10–1.85)
Continuity of care	Low (<50%)	1.00 (ref)	1.00 (ref)	1.00 (ref)	1.00 (ref)
	High (= >50%)	1.79 (1.40–2.30)	2.15 (1.61–2.87)	1.49 (1.20–1.85)	1.61 (1.27–2.04)
Receipt of other childhood vaccinations	No	0.05 (0.03–0.08)	0.04 (0.03–0.07)	n/a	n/a
	Yes	1.00 (ref)	1.00 (ref)	n/a	n/a
**Mother’s characteristics**					
Recent immigrant	No	1.00 (ref)	1.00 (ref)	1.00 (ref)	1.00 (ref)
	Yes	1.38 (0.98–1.95)	1.47 (1.05–2.04)	0.88 (0.64–1.20)	0.82 (0.60–1.12)
Maternal age at first pregnancy	0–24	0.98 (0.82–1.18)	1.23 (1.02–1.47)	0.65 (0.54–0.78)	0.71 (0.58–0.86)
	25–34	1.00 (ref)	1.00 (ref)	1.00 (ref)	1.00 (ref)
	35+	1.07 (0.85–1.35)	1.01 (0.81–1.27)	0.92 (0.73–1.15)	0.88 (0.70–1.11)
Maternal influenza vaccination in year following delivery	No	1.00 (ref)	1.00 (ref)	n/a	n/a
	Yes	1.86 (1.42–2.44)	1.55 (1.24–1.93)	n/a	n/a
Number of children at time of birth of cohort child (including cohort child)	1	1.77 (1.43–2.18)	1.62 (1.30–2.03)	1.29 (1.03–1.62)	1.16 (0.91–1.49)
	2	1.46 (1.16–1.84)	1.31 (1.04–1.65)	0.99 (0.79–1.26)	0.92 (0.73–1.16)
	3+	1.00 (ref)	1.00 (ref)	1.00 (ref)	1.00 (ref)
**Physician’s characteristics**					
Physician’s sex	Female	1.00 (ref)	1.00 (ref)	1.00 (ref)	1.00 (ref)
	Male	0.68 (0.48–0.97)	0.84 (0.59–1.19)	0.83 (0.62–1.11)	0.91 (0.67–1.23)
Rural practice	No	1.00 (ref)	1.00 (ref)	1.00 (ref)	1.00 (ref)
	Yes	0.95 (0.54–1.67)	1.36 (0.82–2.26)	1.72 (1.13–2.63)	2.08 (1.29–3.35)
Foreign trained	No	1.00 (ref)	1.00 (ref)	1.00 (ref)	1.00 (ref)
	Yes	1.32 (0.79–2.21)	1.62 (0.86–3.06)	0.51 (0.35–0.75)	0.60 (0.39–0.92)
Graduation decade	1960-70s	1.00 (ref)	1.00 (ref)	1.00 (ref)	1.00 (ref)
	1980s	0.63 (0.35–1.15)	0.71 (0.33–1.51)	0.71 (0.41–1.21)	0.70 (0.40–1.21)
	1990s	0.63 (0.35–1.16)	0.44 (0.20–0.94)	0.96 (0.58–1.58)	0.67 (0.40–1.15)
	2000s	0.74 (0.43–1.26)	0.52 (0.24–1.13)	1.08 (0.68–1.72)	0.69 (0.40–1.20)
Total number of patients rostered	100-unit increase	0.96 (0.93–0.98)	0.91 (0.88–0.94)	0.96 (0.94–0.98)	0.94 (0.91–0.97)

^1^ Characteristics ‘Receipt of other childhood vaccinations’ and ‘Maternal influenza vaccination in year following delivery’ not assessed in multivariable regression analysis for series completion.

## Discussion

Despite the absence of a physician billing code specific to rotavirus vaccine and the challenges of timely assessment of infant vaccine programs using school-based coverage methods, we were able to conduct a detailed coverage assessment in Ontario by using family physician EMRs. Rotavirus vaccine uptake (series completion) increased each year of the first three years of the program from 73% to 84%, with excellent compliance with age-based dosing guidelines. Linkage to health administrative datasets allowed for factors associated with series initiation and completion to be identified.

Coverage estimates over Ontario’s first three program years are comparable to the early years of program implementation in other large Canadian provinces [32,33]. British Columbia implemented a routine RV program in 2012 and provincial two-dose coverage was estimated to be 70% and 75% among two-year olds in 2014 and 2015, respectively [32]. Two-dose coverage in Quebec, which implemented its routine program in November 2011, was 78% among 2-year old and 86% among 15-month old children when assessed as part of a routine, ongoing coverage survey in 2014 [33]. Higher coverage estimates (>90%) have been observed in Prince Edward Island where infant immunizations are delivered exclusively by public health nurses to the annual birth cohort of approximately 1400 children [34]. All publicly-funded programs implemented to date in Canada use RV1. A Canadian coverage target has not yet been set; not all jurisdictions currently offer rotavirus as part of their publicly funded immunization schedule.

In contrast to other childhood vaccines where un- or under-immunized children can get caught up at a later age, rotavirus vaccines have age-based scheduling recommendations due to a possible age-related association between intussusception and rotavirus vaccination [35]. Our assessment found that compliance with age-based recommendations was excellent; among the 6,527 children who initiated the series during the public program, nearly all children received the first dose before 20 weeks and >99% received the final dose before 32 weeks of age, reflecting NACI’s advice. US investigators have examined compliance with the Advisory Committee on Immunization Practices (ACIP) and product guidelines in distinct groups of infants (i.e. privately-insured infants, Medicaid recipients, separately) rather than through a population-based assessment. In addition, investigators have typically combined adherence with dosing guidelines and series completion into one measure, which reduces the proportion of children assessed as compliant [36–39]. We were not able to identify any Canadian literature that examined compliance with NACI’s rotavirus schedule recommendations.

Identifying and understanding health equity gradients in rotavirus vaccine coverage is an important aspect of program evaluation. We found no association between series initiation or completion and neighbourhood income quintile, an important finding for a system with publicly-funded immunization delivery. Our findings are in alignment with a recent study from a region in the province of Quebec that examined rotavirus coverage by neighbourhood-level characteristics such as unemployment, low income households, households with single mothers, and households with mothers without a high school diploma. They found no significant difference in coverage by any of these measures of socioeconomic status [40]. However, it should be noted that other studies utilizing administrative data in Ontario have found an association between childhood vaccine uptake and neighbourhood income quintile, in contrast to our findings [20,21].

We did not have access to information on children’s ethnicity but were able to explore uptake among infants in newcomer families. Ontario has an ethnically diverse population with approximately 28% of residents born outside of Canada [41]. We found that infants of mothers who had immigrated to Canada within five years of the cohort child’s birth were more likely to initiate the series (aOR = 1.47; 95%CI, 1.05–2.04), consistent with other studies demonstrating a high degree of vaccine acceptance among new Canadians [42,43]. We are unable to explain the observation that children receiving care from a physician who trained outside of Canada were less likely to complete the series. This factor was not significant for series initiation. The proportion of physicians who trained outside of Canada and participate in EMRALD is relatively small and we strongly suggest caution in generalizing this finding to all physicians trained outside of Canada who comprise approximately one-third of practicing physicians in Ontario.

The 2014 CIC rotavirus vaccine recommendations [25] identified analyses of coverage among premature infants as a research priority. Preterm infants are at highest risk for severe rotavirus gastroenteritis [44] and may exceed the age limit recommended for timely administration of the first dose if rotavirus immunization is deferred until hospital discharge [45], which can result in reduced coverage among these vulnerable infants [46]. We found no association between series initiation or completion and prematurity or low birthweight. Although 6.2% of the study cohort eligible for the public program was born preterm (<37 weeks), our inclusion criterion of having an EMR start date within 6 weeks of birth would exclude very preterm infants requiring longer hospital admissions.

Finally, this study re-affirmed previously noted associations for immunization uptake such as the receipt of other routine childhood vaccinations [26,39], having no or fewer siblings [26,27], and attitudinal factors [31], which we assessed by examining maternal influenza vaccine uptake. It also confirmed the importance of health service delivery factors including the importance of continuity of care for immunization delivery [29] and that busy, large physician practices may be sub-optimal for coverage [30].

This study represents the first coverage assessment of rotavirus vaccine to be carried out in Canada’s largest province using a collection of comprehensive family physician EMRs. If immunization information had not been accessed in EMRALD, the first rotavirus coverage assessment would have been delayed until following the 2019–20 school year, when the first age-eligible cohort will be 7 years-of-age, the typical milestone used for coverage assessment in Ontario [47]. Despite these strengths, the reliance on EMR records poses some data quality caveats as the accuracy of our coverage estimate is dependent on the completeness of vaccine administration documentation within EMRALD. However, poor data quality would most likely underestimate vaccine coverage and we found relatively high vaccine uptake and the validation of our search methods suggest that we are unlikely to have missed immunization events. Furthermore, a previous validation study found that EMRALD data was more complete (i.e., contained more immunization data) compared to billing claims [17]. Finally, EMRALD is a voluntary sample of Ontario family physicians. Our study population was similar to the 2013 Ontario birth cohort in most respects, but the study physicians identified through EMRALD were younger, more likely to be female and less likely to have completed their medical training outside of Canada. These physician characteristics likely reflect the characteristics of physicians who have adopted EMR software within their practices and trends in the family physician workforce and it is difficult to comment on the extent to how these differences might influence the generalizability of our findings to the Ontario population. To address this question, a comparison of rotavirus coverage assessed using different data sources (EMRALD versus school-based coverage surveillance) will be feasible in 2020 once eligible children reach the age of routine coverage assessment in Ontario. Finally, our dataset on newcomers only includes immigrants who land in Ontario, and not those whose original point of entry within Canada is another province. This may have under-estimated the proportion of children we identified as having a newcomer mother.

## Conclusions

Rotavirus vaccine uptake increased in the three years following the program’s launch in Ontario. Several maternal/family and physician characteristics were associated with series initiation and completion. This assessment demonstrates the usefulness of EMR data for evaluating vaccine coverage prior to school-entry in Ontario. Our ability to link EMR data to health administrative datasets generated important insights into factors associated with initiation or completion (i.e. continuity of care, roster size, practice location) which should be explored in future research and considered within potential program supports.

## Supporting information

S1 Appendix Search terms used to identify rotavirus vaccines in EMRALD. (DOCX)

S2 Appendix Sensitivity analysis to determine completeness of EMR search strategy to identify rotavirus immunization events. (DOCX)

S3 Appendix Sensitivity analyses on vaccine initiation and full series coverage estimates, by birth cohort and program status, using different criteria for the number of visits to an EMRALD physician within the first year of life exclusion criterion. (DOCX)
