# Supplementary material for: Rotavirus vaccine coverage and factors associated with uptake using linked data: Ontario, Canada
Source: PLoS One. 2018 Feb 14;13(2):e0192809. doi: 10.1371/journal.pone.0192809 (PMC5812625; doi:10.1371/journal.pone.0192809)
Supplement: S1 Appendix — (DOCX) [file pone.0192809.s001.docx]

**S1 Appendix A: Search terms used to identify rotavirus vaccines in EMRALD**

'ROTARIX'

'ROATRIX'

'ROTORIX'

'ROTX'

'Rototrq'

'ROTATEQ'

'ROTATEG'

'ROTA TEQ'

'ROTOTEQ'

'ROTATEC'

'ROTATAQ'

'ROTO VIRUS'

'ROTAVIRUS VACCINE'

'ROTAVIRIS VACCINE’

'ROTRUS VACCINE'

'ROTA VACCINE'

'ROTA%VACCINE'

'ROT%VIRUS%VAC'^1^

^1^The % in the last search term is a wild card character to identify additional spelling errors
