# Supplementary material for: Rotavirus vaccine coverage and factors associated with uptake using linked data: Ontario, Canada
Source: PLoS One. 2018 Feb 14;13(2):e0192809. doi: 10.1371/journal.pone.0192809 (PMC5812625; doi:10.1371/journal.pone.0192809)
Supplement: S2 Appendix — (DOCX) [file pone.0192809.s002.docx]

**Appendix B: Sensitivity analysis to determine completeness of EMR search strategy to identify rotavirus immunization events**

To assess the completeness of our search methodology to identify rotavirus immunization events, we identified 6,255 patients who had no rotavirus immunizations identified through our standard methods from the initial EMRALD cohort of children before study exclusions (n=13,534) and searched all areas of their electronic chart using the search term ‘%rota%’. A total of 1,208 patients had a progress note with this search term. The progress notes were then reviewed manually for a subset of these records (5% random sample, n=62). For seven patients the search term identified a word other than rotavirus (i.e. rotation). Among the remaining 55 patients, the progress note described rotavirus vaccine information and counselling (n=49) or documented a discussion in regards to vaccine refusal (n=6). There were no instances where the progress note clearly described rotavirus vaccine administration.
