# Supplementary material for: Rotavirus vaccine coverage and factors associated with uptake using linked data: Ontario, Canada
Source: PLoS One. 2018 Feb 14;13(2):e0192809. doi: 10.1371/journal.pone.0192809 (PMC5812625; doi:10.1371/journal.pone.0192809)
Supplement: S3 Appendix — (DOCX) [file pone.0192809.s003.docx]

**Appendix C: Sensitivity analyses on vaccine initiation and full series coverage estimates, by birth cohort and program status, using different criteria for the number of visits to an EMRALD physician within the first year of life exclusion criterion.**

**Table 5: Including those with only one visit to the EMRALD physician (n=13,084)**

| Program status | Birth cohort | Cohort size | Proportion of children who initiated the rotavirus vaccine series^1^  (with 95% CI) | Proportion of initiators who completed series^2^ (all doses considered)  (with 95% CI) | Coverage estimate for series completion^2^ (all doses considered)  (with 95% CI) |
| --- | --- | --- | --- | --- | --- |
| Private availability | Jan. 1-Dec. 31, 2008 | 1282 | 4.8%  (3.6%, 6.0%) | 61.3%  (49.2%, 73.4%) | 2.9%  (2.0%, 3.8%) |
| Private availability | Jan. 1-Dec. 31, 2009 | 1763 | 7.9%  (6.6%, 9.2%) | 63.3%  (55.3%, 71.3%) | 5.0%  (4.0%, 6.0%) |
| Private availability | Jan. 1-Dec. 31, 2010 | 2204 | 10.6%  (9.3%, 11.9%) | 65.4%  (59.3%, 71.5%) | 6.8%  (5.7%, 7.9%) |
| Program year 1 | Aug. 1 2011-July 31, 2012 | 2855 | 82.3%  (80.9%, 83.7%) | 87.1%  (85.7%, 88.5%) | 71.5%  (69.8%, 73.2%) |
| Program year 2 | Aug. 1 2012-July 31, 2013 | 2926 | 87.0%  (85.8%, 88.2%) | 87.7%  (86.4%, 89.0%) | 76.2%  (74.7%, 77.7%) |
| Program year 3 | Aug. 1 2013-July 31, 2014 | 2054 | 90.3%  (89.0%, 91.6%) | 91.7%  (90.4%, 93.0%) | 82.6%  (81.0%, 84.2%) |

Notes:

^1^Receipt of at least one dose of rotavirus vaccine (RV1 or RV5).

^2^Series completion required two doses if all the doses were RV1. Three doses were required if all doses were RV5 or if a mix of products (RV1 and RV5) were used.

**Table 6: No exclusions based on the number visits to the EMRALD physician (n=13,467)**

| Program status | Birth cohort | Cohort size | Proportion of children who initiated the rotavirus vaccine series^1^  (with 95% CI) | Proportion of initiators who completed series^2^ (all doses considered)  (with 95% CI) | Coverage estimate for series completion^2^ (all doses considered)  (with 95% CI) |
| --- | --- | --- | --- | --- | --- |
| Private availability | Jan. 1-Dec. 31, 2008 | 1321 | 4.7%  (3.6%, 5.8%) | 61.3%  (49.2%, 73.4%) | 2.8%  (1.9%, 3.7%) |
| Private availability | Jan. 1-Dec. 31, 2009 | 1827 | 7.6%  (6.4%, 8.8%) | 63.3%  (55.3%, 71.3%) | 4.8%  (3.8%, 5.8%) |
| Private availability | Jan. 1-Dec. 31, 2010 | 2260 | 10.4%  (9.1%, 11.7%) | 65.1%  (59.0%, 71.2%) | 6.6%  (5.6%, 7.6%) |
| Program year 1 | Aug. 1 2011-July 31, 2012 | 2935 | 80.9%  (79.5%, 82.3%) | 86.9%  (85.5%, 88.3%) | 70.2%  (68.5%, 71.9%) |
| Program year 2 | Aug. 1 2012-July 31, 2013 | 3021 | 85.2%  (83.9%, 86.5%) | 87.6%  (86.3%, 88.9%) | 74.5%  (72.9%, 76.1%) |
| Program year 3 | Aug. 1 2013-July 31, 2014 | 2103 | 88.8%  (87.5%, 90.1%) | 91.8%  (90.4%, 93.0%) | 81.3%  (79.6%, 83.0%) |

Notes:

^1^Receipt of at least one dose of rotavirus vaccine (RV1 or RV5).

^2^Series completion required two doses if all the doses were RV1. Three doses were required if all doses were RV5 or if a mix of products (RV1 and RV5) were used.
